# The clinical effect of recombinant human brain natriuretic peptide on asymptomatic peri-procedural myocardial injury after percutaneous transluminal coronary angioplasty

**DOI:** 10.1038/s41598-020-72710-3

**Published:** 2020-09-28

**Authors:** Ling Liang, Rong Tang, Qiang Xie, Junyu Han, Weihua Li

**Affiliations:** 1 Department of Cardiology, The First Affiliated Hospital of Xiamen University, Xiamen, Fujian People’s Republic of China; 2 Department of Cardiology, First Clinical Medical College, Fujian Medical University, Fuzhou, People’s Republic of China

**Keywords:** Cardiology, Cardiac device therapy

## Abstract

To determine the effect of intravenous injection of recombinant human brain natriuretic peptide (rhBNP) on lowering the incidence of asymptomatic peri-procedural myocardial injury (PMI) in patients who underwent coronary stent implantation. In this retrospective observational study, data pooled from a tertiary hospital electronic medical records were used to quantify the troponin enzyme change after patients with coronary artery disease (CAD) were pretreated with rhBNP infusion one day prior to percutaneous coronary intervention (PCI). The primary end point was to analyze the incidence of the elevated high-sensitivity cardiac troponin I serum levels above the upper normal limit after PCI. A total of 156 CAD patients were enrolled into rhBNP group (n = 76) and control group (n = 80). The incidence of asymptomatic PMI was 33% in the rhBNP group versus 51% in the control group (*P* = 0.02) after PCI. At eight months, the incidences of composite endpoints were 25.3% in the control group and 13% in the rhBNP group (difference, 12.3 percentage points; 95% confidence interval (CI), 0.197 to 1.048; *P* = 0.061). There were 7 visits in the rhBNP group and 15 visits in the control group for recurrent angina (difference, 10 percentage points; 95% CI 0.168–1.147; *P* = 0.087). A time-to-event analysis of the composite clinical endpoints and the recurrent angina between the control group and rhBNP group showed that the hazard ratios were 2.566 (95% CI 1.187–5.551; *P* = 0.017) and 2.607 (95% CI 1.089–6.244; *P* = 0.032) respectively. The decreased incidence of asymptomatic PMI after PCI and the reduced episodes of recurrent angina at eight months follow-up were associated with the administration of rhBNP infusion prior to PCI.

## Introduction

Although percutaneous coronary intervention (PCI) has been very successful due to the advances in technologies and equipment, peri-procedural myocardial injury (PMI), the change of myocardial biomarkers after angioplasty, is common with the incidence of 35%^1^. PMI is associated with adverse clinical events and increased mortality^2–6^. The proposed pathogenesis of PMI involves the distal embolism from the atherosclerosis plaque debris and microvascular thrombosis, acute occlusion of the side-branch, as well as the microvascular spasm^7–9^.

Recombinant human brain natriuretic peptide (rhBNP) is effective in the treatment of acute or chronic heart failure^10–12^. In addition, an animal study showed that BNP activates the membrane-bound guanylyl cyclase-A receptor, resulting in the accumulation of intracellular cyclic guanine monophosphate (cGMP) in target tissues. The accumulation of cGMP mediates the vascular relaxation of smooth muscles^13^. In clinical studies, human BNP infusion exerts vasodilatory effects on epicardial arteries, coronary conductivity and arterial resistance^14,20^. The evidences suggest that BNP dilates coronary arteries, improves arterial resistance and effectively protects against myocardial embolism. The objectives of our study are to determine the incidence of asymptomatic PMI and to observe clinical adverse events during the follow-up after rhBNP pretreatment in CAD patients undergoing PCI.

## Methods

### Patient selection

From April 1, 2016 to December 31, 2016, we reviewed our hospital electronic medical records and consecutively included CAD patients who were candidates for PCI and were pretreated with rhBNP injection or conventional treatment. The following patients were excluded from this study: (1) cardiogenic shock; (2) acute ST-segment elevation myocardial infarction or non-ST-segment elevation myocardial infarction; (3) left main stenosis; (4) PCI failures; (5) severe congenital or valvular heart disease requiring surgery; (6) baseline abnormal high-sensitivity cardiac troponin I (hsCTnI); (7) multi-vessels stenosis candidates for bypass; and (8) estimated glomerular filtration rate < 30 mL/min/1.73 mm^2^; (9) no hsCTNI test results after PCI.

### Research design and PCI procedure

Dual antiplatelet coagulation agents (aspirin 300 mg and clopidogrel 600 mg) and cholesterol lowering drugs were given the days before PCI. The interventional group received lyophilized rhBNP infusion one day prior to PCI with a loading dose of 1.5 μg/kg and maintained of 0.0075 μg kg^−1^ min^−1^ (dose was adjusted according to blood pressure) and the infusion continued for the rest days of hospitalization. The control group received a conventional treatment.

All PCI procedures were performed according to the present guidelines^15^. Weight-adjusted unfractionated heparin or bivalirudin was administrated before the placement of the stent and discontinued at the end of the procedure. After PCI, weight-adjusted low-molecular-weight heparin was injected subcutaneously during hospitalization. Clopidogrel 75 mg once per day for at least 12 months and lifelong 100 mg aspirin once a day treatment were prescribed to all patients after PCI. To evaluate the effect of rhBNP infusion on the reduction of asymptomatic PMI incidence, the hsCTnI serum levels were measured before PCI and 24 h after the procedure using a radioimmunoassay analyzer. The reference range for hsCTnI was 0–0.026 ng/ml in our hospital. The follow-up time started from the first visit date after discharge and ended on December 31, 2016. The informed consents were obtained from all patients.

### Study end point

The primary end point was the asymptomatic PMI after PCI. The asymptomatic PMI was diagnosed by the elevated hsCTnI serum levels above the UNL within 24 h after the procedure without clinical symptoms such as chest pain or dyspnea^16^. The secondary end point included a composite of cardiac death, non-fatal acute myocardial infraction, target-vessel revascularization and recurrent angina.

### Statistical analysis

All data were obtained from the electronic medical database and entered in a spreadsheet to do the data calculation. Qualitative parameters were summarized by using the absolute and percentage numbers. Quantitative parameters were summarized by using the means ± standard deviation for normally distributed variables and the median with interquartile range for the non-normally distributed ones. The comparison between continuous variables in both groups were done with the Student’s *t* test for normally distributed values. The Mann–Whiteney *U* test were applied as non-normally distributed quantitative values were compared between the two groups. The Pearson’s χ^2^-test was used to analyze the difference between the counts and percentages in both groups, and the Fisher exact test was applied if the expected frequency was less than five. A survival analysis was conducted for the clinic outcomes. This approach examined time to first event of clinic visit or hospital admission. The hazard ratios (HRs) and 95% confidence intervals (CIs) were calculated from the results of the Cox hazards analyses. A two-side *P* value less than 0.05 was considered to indicate statistically significant. All statistical analyses were carried out using the SPSS statistical software (version 24.0; SPSS Inc., Chicago, Illinois, USA).

### Ethical approval

All procedures performed in studies involving human participants were in accordance with the ethical standards of the Ethics Committee of The First Affiliated Hospital of Xiamen University and with the 1964 Helsinki declaration and its later amendments or comparable ethical standards. This study was approved by the Medical Research Ethics Committee of The First Affiliated Hospital of Xiamen University.

## Results

### Clinical data

**Figure 1:**
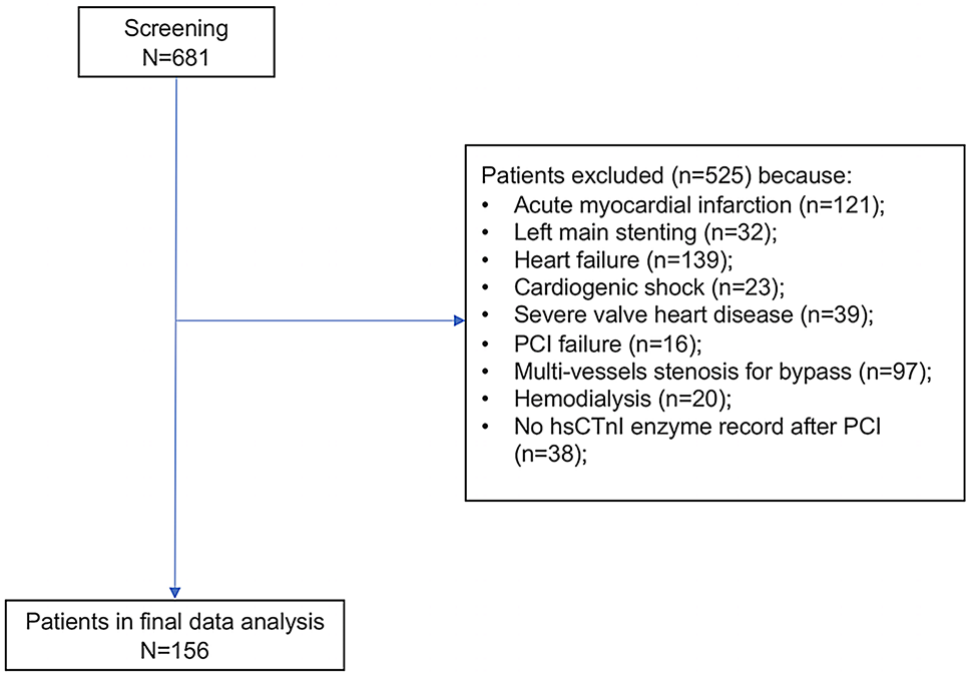
Flow diagram of the included patients.

681 patients were initially screened and 525 patients were excluded according to the inclusion and exclusion criteria (see Fig. 1). Totally, 156 patients with CAD, including stable angina, asymptomatic ischemic coronary artery disease and unstable angina were enrolled.

Patients were observed and divided into the rhBNP group (n = 76) and the control group (n = 80). The baseline demographic and clinical characteristics of patients in both groups were summarized in Table 1. There was no difference in gender, risk factor distribution, blood creatinine level, mild aortic valve calcification or hsCTnI level before PCI between two groups. No significant differences were found in the baseline of procedural features (Table 2).

**Table 1:** Baseline Clinical Characteristics of patients.

Variables	N (%,or interquartile range)		*P* value
	Control N = 80	rhBNP N = 76	
Unstable angina	46 (58)	50 (66)	0.287
Male	55 (69)	49 (64)	0.571
age	64.76 ± 1.13	65.64 ± 1.17	0.595
Creatinine (µmol/L)	75.3 ± 1.87	72.93 ± 1.72	0.25
Diabetes	24 (30)	16 (21)	0.201
Hypertension	44 (55)	40 (53)	0.767
Smoking	16 (20)	18 (24)	0.577
AVC	18 (32)	21 (28)	0.459
BNP (pg/ml)	21.1 (9.93,52.28)	21.3 (1.58, 44.08)	0.673
TC (mmol/L)	4.37 ± 0.12	4.59 ± 0.15	0.235
TG (mmol/L)	1.48 (0.98, 1.96)	1.23 (0.91, 1.79)	0.201
LDL (mmol/L)	2.52 ± 0.1	2.62 ± 0.12	0.524
hsCTNI (ng/ml)	0.003 (0.002–0.007)	0.002 (0.001–0.006)	0.054

AVC, aortic valve calcification; BNP, brain natriuretic peptide; hsCTNI, high-sensitivity cardiac troponin I; LDL, low density lipoprotein; LVEF, left ventricular ejection fraction; TC, total cholesterol; TG, triglyceride.

**Table 2:** Angiographic and Interventional characteristics.

Variables	N (%, or interquartile range)		*P* value
	Control n = 80	rhBNP n = 76	
**Target artery**			
LAD	37 (46)	43 (57)	0.197
LCX	10 (13)	10 (13)	0.902
RCA	30 (38)	23 (30)	0.34
Bifurcation	6 (8)	4 (5)	0.747
Post dilation	22 (28)	22 (29)	0.841
**Number of stents implanted**			
1	59 (74)	63 (83)	0.167
2	21 (26)	13 (17)	0.167
Multivessel disease	25 (31)	21 (28)	0.62
**Anticoagulation**			
Bivalirudin	27 (34)	28 (37)	0.686
Unfractionated heparin	53 (66)	48 (63)	0.686
Stent length (mm)	24 (18–29)	20 (18–28)	0.084
Stent diameter (mm)	3.0 (2.75–3.5)	3.0 (2.75–3.5)	0.631
Acute branch occlusion	0	0	–

LAD, left anterior descending artery; LCX, left circumflex artery; RCA, right coronary artery.

### The primary end point

**Figure 2:**
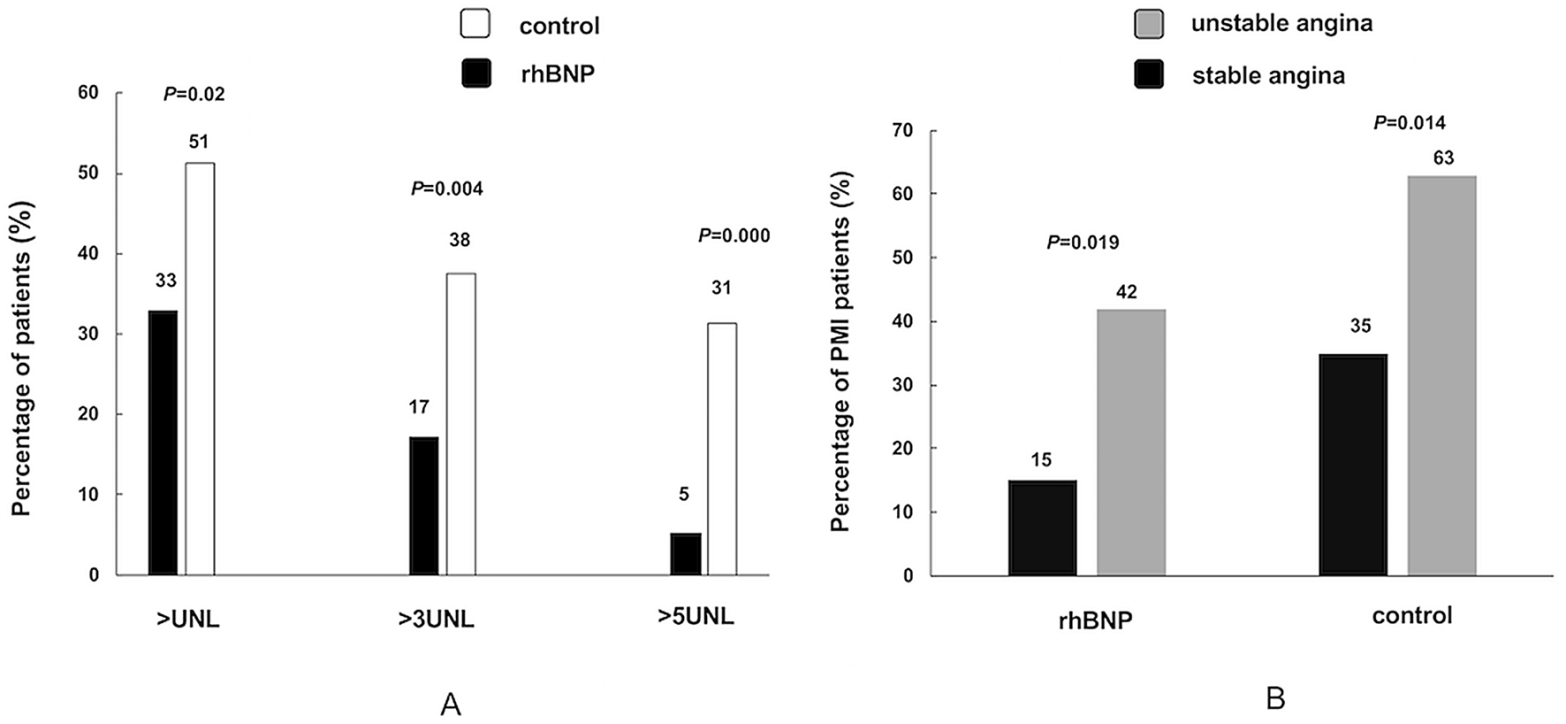
The comparison of percentages of PMI between rhBNP group and control group. (**A**) The different percentages of hsCTnI level more than the UNL, three times the UNL and five times the UNL between rhBNP group and control group. (**B**) The comparison of the percentage of PMI between the unstable angina patients and stable angina patients in both groups. PMI: peri-procedural myocardial injury; rhBNP: recombinant human brain natriuretic peptide; UNL: upper normal limit.

After PCI, the percentages of asymptomatic PMI were 33% in the rhBNP group and 51% in the control group (*P* = 0.02). The percentage of hsCTnI levels more than three times the UNL was significantly decreased by the rhBNP infusion as compared to the conventional treatment (17% vs. 38%, *P* = 0.004). The hsCTnI levels more than five times the UNL occurred in 5% in the rhBNP group and in 31% in the control group (*P* = 0.000). In the rhBNP group, the incidence of PMI in the unstable angina was approximately three times higher than the incidence in the stable angina (*P* = 0.019). In the control group, there were 29 (63%) PMI cases in the unstable angina and 12 (35%) PMI cases in the stable angina (*P* = 0.014). In the unstable angina subgroup analysis, the incidences of PMI were 42% in the rhBNP group and 63% in the control group respectively (*P* = 0.038). Although no significant difference was observed between two groups in the stable angina subgroup (*P* = 0.084), it appeared that PMI occurrence in the rhBNP group was lower than that in the control group (Fig. 2). However, the total hsCTnI serum levels after PCI did not change significantly between rBNP group (median, 0.018 ng/mL; interquartile range 0.009–0.0515) and control group (median 0.03 ng/mL, interquartile range 0.012–0.176; *P* = 0.083).

### Major adverse cardiac events

No sudden severe blood pressure decrease (systolic pressure below 90 mmHg) was observed during the hospitalization. At eight months follow-up, non-fatal acute myocardial infraction or cardiac death occurred.

**Figure 3:**
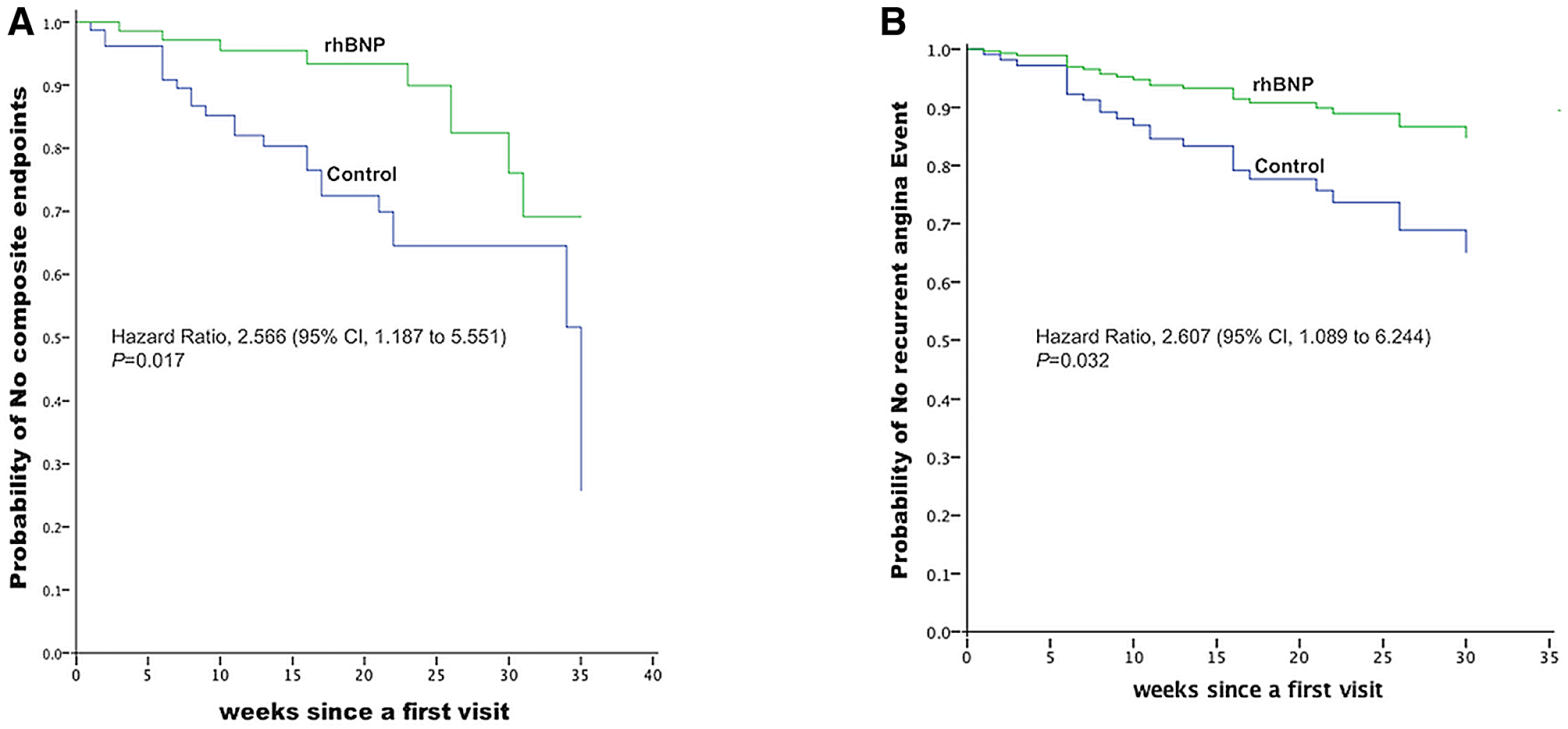
Kaplan–Meier curves for composite endpoints (**A**) and recurrent angina (**B**). Composite endpoints include: cardiac death, non-fatal acute myocardial infraction, target-vessel revascularization and recurrent angina. rhBNP: recombinant human brain natriuretic peptide.

The incidences of composite endpoints were 25.3% in the control group and 13% in the rhBNP group (difference, 12.3 percentage points; 95% CI, 0.197 to 1.048; *P* = 0.061). The survival analysis of composite endpoints indicated the hazard ratio was 2.566 (95% CI 1.187 to 5.551; *P* = 0.017).Target-vessel revascularization occurred in five patients (6.3%) in the control group and three patients (4%) in the rhBNP group (difference, 2.3 percentage points; 95% CI 0.142 to 2.676; *P* = 0.72). A time-to-event analysis of the target-vessel revascularization showed a similar result (hazard ratio HR, 1.646; 95% CI, 0.391to 6.929; *P* = 0.497). In terms of the recurrent angina, there were 15 cases in the control group versus 7 cases in the rhBNP group (difference, 10 percentage points; 95% CI 0.168 to 1.147; *P* = 0.087) (Table 3). An additional survival analysis of the recurrent angina events indicated that the hazard ratio was 2.607 (95% CI, 1.089 to 6.244; *P* = 0.032) (Fig. 3).

**Table 3:** Clinical Outcomes at eight months.

Adverse Event	rhBNP (n = 75) Control (n = 79) no./total no.(%)		Difference (95% CI)	*P* value
Composite endpoints	10/75 (13)	20/79 (25.3)	− 12.3 (0.197–1.048)	0.061
Target-vessel revascularization	3/75 (4)	5/79 (6.3)	− 2.3 (0.142—2.676)	0.72
Recurrent angina	7/75 (9)	15/79 (19)	− 10 (0.168—1.147)	0.087

CI: confidence interval; rhBNP: recombinant human brain natriuretic peptide.

## Discussion

This study retrospectively evaluated the protective effects of the rhBNP infusion on asymptomatic PMI in CAD patients undergoing stent implantation during hospitalization and the clinical outcomes in the follow-up. The total incidence of asymptomatic PMI was decreased in the rhBNP group as compared with that in the control group. The incidence of PMI in the unstable angina was approximately two times higher than the incidence in the stable angina. The rhBNP treatment protective effect was exerted on the unstable angina rather than the stable angina. Although there was no significant difference between the rhBNP group and the control group in eight-month follow-up of composite endpoints and recurrent angina, the time-to-even analysis result indicated that the rhBNP regimen decreased composite clinical endpoints and alleviated patients angina episodes.

The cardiac troponin is a more specific and sensitive biomarker than creatine phosphokinase MB isoenzyme (CK-MB) for the accuracy of myocardial injury detection^17,18^. One meta-analysis pooled 20 studies over a 19-year period and concluded that troponin elevation post-PCI increased 1.35-fold mortality^1^. In this research, we found that the incidence of PMI in the study was 51% in the control group, higher than 35% reported by other researches. One reason of the higher incidence of PMI in our study was that hsCTnI assays allowed more precise quantification of cardiomyocytes injury around the 99th percentile^19^. Another reason was that we included patients with unstable angina accounting for more than 50% in this study, which could increase the risk of PMI development^20–22^.

The various potential pathophysiological factors are likely to explain the protective effects of rhBNP infusion on PMI. Exogenous rhBNP is a 17-cyclic polypeptide consisting of 32 amino acid residues and has been clinically used to treat heart disease for decades. RhBNP improves kidney blood flow and increases glomerular filtration rate to expel body volume for heart failure^23^. One clinical trial shows that this treatment conserves left ventricular ejection function after acute myocardial infarction with primary PCI^24^. Additionally, rhBNP injection treatment reduces pulmonary artery wedge pressure, dilates coronary artery and increases coronary blood perfusion^25,26^. As for the acute myocardium ischemia revascularization, rhBNP regimen enhances anti-myocardial ischemia and anti-hypoxia capacity^27^. The early application of rhBNP to treat acute myocardial infarction patients undergoing emergent PCI procedure has not only improved myocardial perfusion, limited myocardial infarct size and ameliorated myocardial dysfunction, but also decreased the area under the curve (AUC) of both CK-MB and TNI^28^. Another clinical study suggests that the administration of rhBNP significantly improves the resilience of the local infarcted myocardium no matter when the rhBNP regimen began (prior to or after PCI)^29^. In this study, the rhBNP treatment reduced the risk of PMI development, especially among the unstable angina patients, which could be related to the coronary artery dilation and the microvascular embolism decrease.

### Study limitations

Because this study was retrospective cohort observation, the selection bias could not be excluded. The longer follow-up term was needed to determine the long-term clinical benefits. Recurrent angina visits were judged only by different physicians and a standardized criteria for recurrent angina were needed to decrease variation.

## Conclusion

The significantly decreased incidence of asymptomatic PMI was associated with rhBNP infusion regimen. The risk of developing recurring angina episodes was reduced at eight-month follow-up. Monitoring of hsCTnI post-PCI for the unstable angina patients is highly recommended. Active medication prophylaxis could improve clinical outcomes among unstable angina patients after PCI.
